# Identification of a *Solanum pennellii* Chromosome 4 Fruit Flavor and Nutritional Quality-Associated Metabolite QTL

**DOI:** 10.3389/fpls.2016.01671

**Published:** 2016-11-09

**Authors:** Zhongyuan Liu, Saleh Alseekh, Yariv Brotman, Yi Zheng, Zhangjun Fei, Denise M. Tieman, James J. Giovannoni, Alisdair R. Fernie, Harry J. Klee

**Affiliations:** ^1^Horticultural Sciences Department, Genetics Institute, University of Florida, GainesvilleFL, USA; ^2^Max Planck Institute of Molecular Plant PhysiologyPotsdam-Golm, Germany; ^3^Department of Life Sciences, Ben-Gurion University of the NegevBeersheba, Israel; ^4^US Department of Agriculture–Agricultural Research Service Robert W. Holley Center for Agriculture and Health, Cornell University, IthacaNY, USA; ^5^Robert W. Holley Center for Agriculture and Health, Cornell University and US Department of Agriculture–Agricultural Research Service, IthacaNY, USA

**Keywords:** flavor, fruit ripening, metabolome, transcriptome, IL4-4

## Abstract

A major resource for tomato quality improvement and gene discovery is the collection of introgression lines (ILs) of cultivated *Solanum lycopersicum* that contain different, defined chromosomal segments derived from the wild tomato relative, *S. pennellii.* Among these lines, IL4-4, in which the bottom of *S. lycopersicum* (cv. M82) chromosome 4 is replaced by the corresponding *S. pennellii* segment, is altered in many primary and secondary metabolites, including many related to fruit flavor and nutritional quality. Here, we provide a comprehensive profile of IL4-4 ripe fruit metabolites, the transcriptome and fine mapping of sub-ILs. Remarkably, out of 327 quantified metabolites, 185 were significantly changed in IL4-4 fruit, compared to the control. These altered metabolites include volatile organic compounds, primary and secondary metabolites. Partial least squares enhanced discriminant analysis of the metabolite levels among sub-ILs indicated that a genome region encompassing 20 putative open reading frames is responsible for most of the metabolic changes in IL4-4 fruit. This work provides comprehensive insights into IL4-4 fruit biochemistry, identifying a small region of the genome that has major effects on a large and diverse set of metabolites.

## Introduction

Recent research has highlighted the importance of crop compositional quality for human health (Demmig-Adams and Adams, 2002; Spencer et al., 2005; Martin et al., 2011). Tomato, as one of the most important fruit crops worldwide, represents a valuable source of micronutrients including amino acids, vitamins and antioxidants. Despite its importance as a crop and a major component of the human diet, fruit quality has deteriorated in recent years. In particular, the flavor of modern commercial varieties is generally perceived as poor as breeders have focused most attention on yield. Natural variation in the wild relatives of tomato is a potential source of genetic and biochemical diversity for improvement of flavor and nutrition of the cultivated tomato (Fernie et al., 2006; Schauer et al., 2006; Klee and Tieman, 2013). *Solanum pennellii*, a wild South American relative, has a complete genome sequence (Bolger A. et al., 2014) and a well characterized collection of introgression lines (ILs) (Eshed and Zamir, 1995; Liu and Zamir, 1999; Chitwood et al., 2013). These ILs have been extensively exploited for discovery of fruit quality associated QTLs, including soluble solids content, volatile emissions, pigment biosynthesis as well as primary/secondary metabolite production (Causse et al., 2004; Baxter et al., 2005; Schauer et al., 2006; Tieman et al., 2006; Perez-Fons et al., 2014; Alseekh et al., 2015). However, only a few of the loci affecting fruit chemical composition have been mapped to high resolution using sub-isogenic lines (sub-ILs) (Fridman et al., 2004; Alseekh et al., 2015).

Previous studies of the *S. pennellii* IL population indicated that the bottom of chromosome 4 contains QTLs for many horticulturally important traits including soluble solids content, fruit shape, lycopene content and chemical composition (Yates et al., 2004; Schauer et al., 2006; Tieman et al., 2006; Mathieu et al., 2009; Alseekh et al., 2015). However, the epidermal reticulation phenotype of IL4-4 fruit controlled by *CUTICULAR WATER PERMEABILITY 1* (*CWP1*) is a barrier to utilizing the genetic variation of wild relatives at the bottom of chromosome 4 for improving tomato fruit quality (Hovav et al., 2007). At a fundamental level, the molecular basis for the major effects on such a broad range of fruit metabolites in IL4-4 is an interesting and important challenge.

Identification of metabolite QTLs using metabolite profiling is a powerful tool to understand the complex mechanisms underlying regulation of metabolic pathways. In *Arabidopsis*, Keurentjes et al. (2006) identified more than 4000 metabolite QTLs using untargeted metabolomics methods in a recombinant inbred population derived from two most divergent *Arabidopsis* accessions. The combination of rice metabolic profiling with an ultrahigh-density genetic map greatly accelerated the gene identification and pathway elucidation for metabolites in rice (Gong et al., 2013). A refinement on metabolomic profiling to identify genes underlying important QTLs is construction of transcriptome-enabled regulatory networks, as illustrated in *S. pennellii*-derived ILs (Fei et al., 2011; Chitwood et al., 2013). Comparative analysis of RNA-Seq data from M82 and ILs may reveal the transcriptomic changes regulated by the introgressed genome segment. The combination of metabolite profiling and RNA-Seq can facilitate understanding points of metabolic regulation (Adato et al., 2009) and identification of the causative genes (Chambers et al., 2014).

Here, we performed a large-scale metabolic analysis with a fine-mapped sub-IL population derived from IL4-4 combined with RNA-Seq performed on ripe fruit tissue of IL4-4 and M82. Out of 327 quantified fruit metabolites, 185 were altered in the IL4-4 fruit. Further QTL mapping identified a locus encompassing twenty annotated genes near the bottom of chromosome 4 that is responsible for the overall metabolic changes associated with IL4-4.

## Materials and Methods

### Plant Material and Growth Conditions

Plants used for volatile analysis were grown in the field at Live Oak, FL, USA; Plants used for other metabolites analysis were grown in greenhouses at Golm, Germany. To generate a series of sub-ILs, a total of 688 F2 plants were derived from a cross between IL4-4 and M82. DNA was extracted from each individual and screened for the flanking ends of the introgression with markers C198103-1 and C10. This screen resulted in the isolation of 57 recombinant individuals within the interval. Points of recombination within these sub-ILs were further defined by the markers listed in Supplementary Table S1. Thirty sub-ILs were selected for propagating homozygous seeds in the F3 generation. Ripe fruits from F4 plants were harvested for metabolite profiling.

To validate consistency of chemical profiling across sites, we examined multiple metabolites quantified in both locations. Glucose, fructose, citric, and malic acids were expressed as a ratio of IL4-4 to M82. These numbers were consistent across both locations (Supplementary Table S2).

### Volatile Collection and Analysis

Volatile organic compounds (VOCs) were collected from ripe fruits of sub-ILs R401, R2174, R2075, R100, R434 as well as the parent lines, IL4-4 and M82, grown in randomized, replicated plots. Each biological replicate was a mixture of five to six individual fruit at the red ripe stage. Collection of VOCs was performed as described previously (Tieman et al., 2006). In brief, VOCs were collected from chopped ripe fruits (peel and flesh) during a 1-h period. The VOCs were trapped on SuperQ resin and subsequently eluted with methylene chloride using nonyl acetate as an internal control. The samples were separated on a DB-5 column (Agilent)^1^ and analyzed on an Agilent 6890N gas chromatograph equipped with a flame ionization detector. Retention times compared with known standards and identities of volatile peaks were confirmed by gas chromatography/mass spectrometry (GC/MS) (Agilent 5975 GC/MS)^1^. The list of quantified volatile compounds is presented in Supplementary Table S3.

### Primary Metabolites Profiling

The extraction method was performed as described by Giavalisco et al. (2011). In short, samples were extracted with 1 ml of methanol/methyl tert-butyl ether/water mixture. After incubation in 4°C and sonication for 10 min in an ice-cooled sonic bath, 500 ml of water/methanol mixture was added. This led to the formation of two phases: a lipophilic phase and a polar phase. For the primary metabolites aliquot from the polar phase was collected and dried under vacuum, and the residue was derivatized for 120 min at 37°C (in 50 μl of 20 mg ml-1 methoxyamine hydrochloride in pyridine) followed by a 30 min treatment at 37°C with 50 μl of MSTFA. The GC-MS system used was a gas chromatograph coupled to a time-of-flight mass spectrometer (Pegasus III, Leco). An autosampler system (PAL) injected the samples. Helium was used as carrier gas at a constant flow rate of 2 ml/s and gas chromatography was performed on a 30 m DB-35 column. The injection temperature was 230°C and the transfer line and ion source were set to 250°C. The initial temperature of the oven (85°C) increased at a rate of 15°C/min up to a final temperature of 360°C. After a solvent delay of 180 s mass spectra were recorded at 20 scans s-1 with m/z 70–600 scanning range. Chromatograms and mass spectra were evaluated by using Chroma TOF 1.0 (Leco) and TagFinder 4.0 software (Roessner et al., 2001; Schauer et al., 2005).

Sugars (glucose, fructose) and acids (citric, malic) were quantified in Florida as previously described (Vogel et al., 2010).

### Secondary Metabolites Profiling

For secondary metabolites the rest of polar phase (see above extraction protocol) was dried and residue was suspended in 200 μl of 80% methanol water (80:20). The extracts were then subjected to LC-MS analysis using a high-performance liquid chromatography (HPLC; Surveyor; Thermo Finnigan, USA), coupled to a Finnigan LTQ-XP system (Thermo Finnigan, USA), Metabolite identification and annotation were performed using a combination of standard compounds and tomato metabolomics databases (Moco et al., 2006; Iijima et al., 2008; Tohge and Fernie, 2009, 2010; Rohrmann et al., 2011).

### Lipid Extraction and Analysis

For lipid extraction, the lipophilic phase (see extraction protocol above) was collected and vacuum-dried. Samples were processed using ultra-performance liquid chromatography coupled with Fourier transform mass spectrometry (UPLC-FT-MS, Hummel et al., 2011), on a C8 reverse phase column coupled with an Exactive mass spectrometer (Thermo-Fisher)^2^ in positive and negative ionization mode. Processing of chromatograms, peak detection and integration were performed using REFINER MS^®^ 6.0 (GeneData)^3^. Processing of mass spectrometry data included the removal of the fragmentation information, isotopic peaks, as well as chemical noise. Obtained features (m/z at a certain retention time) were queried against an in-house lipid database for further annotation.

### Brix Value Determination

Five ripe tomato fruits were homogenized in a blender for 30 s and frozen at -80°C until analysis. Samples were thawed and 1.5 mL was centrifuged at 16 000 × *g* for 5 min. The supernatant was used to calculate °Brix with a handheld refractometer (ATAGO N-20, Japan).

### QTL Mapping

To map the metabolite variation in the sub-ILs, a one-way analysis of variance (ANOVA; level of significance set as *p* < 0.01) was used to determine the QTL controlling metabolite content. All lines were compared with M82 and each other. If metabolite level of the line was significantly different from the M82 control (indicated by Dunnett’s *t*-test *p* < 0.01), a line was considered as harboring a QTL. A list of QTLs within the sub-ILs can be found at Supplementary Table S4.

### Heat Map

The heat map was generated with MutiExperiment Viewer 4.0. False color imaging was done on the log2-transformed metabolite data. Metabolite data were the average value of all replicates for each line.

### Statistical Analysis

Unpaired Student’s *t*-test was used for two-sample comparisons. For multiple comparisons, an ANOVA was performed followed by a Newman–Keuls test. The level of significance is indicated in each table and figure. Partial least squares enhanced discriminant analysis (PLS-EDA) was performed by using the Excel add-in Multibase package (Numerical Dynamics, Japan).

### RNA Extraction and RNA-seq

Total RNA was extracted from frozen pericarp tissue of ripe fruits as described in Griffiths et al. (1999). Strand-specific RNA-Seq libraries were constructed using the protocol described in Zhong et al. (2011) and sequenced on the Illumina HiSeq 2000 platform using the single-end mode. Raw RNA-Seq reads were processed using Trimmomatic (Bolger A.M. et al., 2014) to remove adaptor and low quality sequences. RNA-Seq reads were then aligned to the ribosomal RNA database (Quast et al., 2013) using Bowtie allowing three mismatches (Langmead et al., 2009) and the mappable reads were discarded. The resulting high-quality cleaned reads were aligned to the tomato reference genome (The Tomato Genome Consortium, 2012) using TopHat allowing one segment mismatch (Trapnell et al., 2009). Following alignments, raw counts for each tomato gene were derived and normalized to reads per kilobase of exon model per million mapped reads (RPKM). Raw counts were then fed to the DESeq package (Anders and Huber, 2010) for differential expression analysis. Genes with adjusted p values less than 0.05 and fold changes greater than or equal to 2 were identified as differentially expressed genes (DEGs) between IL4-4 and M82.

### SIFT (Sorting Intolerant from Tolerant) Analyses

The deduced amino acid sequences from *S. lycopersicum* and *S. pennellii* were aligned using ClustalW^4^, and polymorphic sequences were submitted to SIFT^5^ to predict the impact of amino acid substitutions on protein function (Kumar et al., 2009).

### Gene Ontology (GO) Enrichment Analysis

Gene ontology enrichment analyses of DEGs were conducted with PANTHER^6^. GO terms for biological process and molecular function were retrieved with functions of this web-tool. The heatmap of metabolism overview was designed using Mapman software (Mapman version 3.0.0). A dot represents the log2 of the RPKM ratio of a transcript between IL4-4 and control M82.

### Taste Panel Analysis

Twenty fruits harvested from R2174, R434, R2075, IL4-4, and M82 were subjected to sensory evaluation. Samples were cut into wedges and labeled with random numbers. Sample taste tests were performed on two occasions with two separate harvests. Each taste panel had 14 panelists. Each sample was rated on a 10-point hedonic scale (1–10, with 10 as like extremely). Taste preference scores are an averaged score of all panelists. Significant differences were calculated using Student’s *T*-test.

## Results

### Metabolite Profiling of IL4-4 Fruit

**FIGURE 1:**
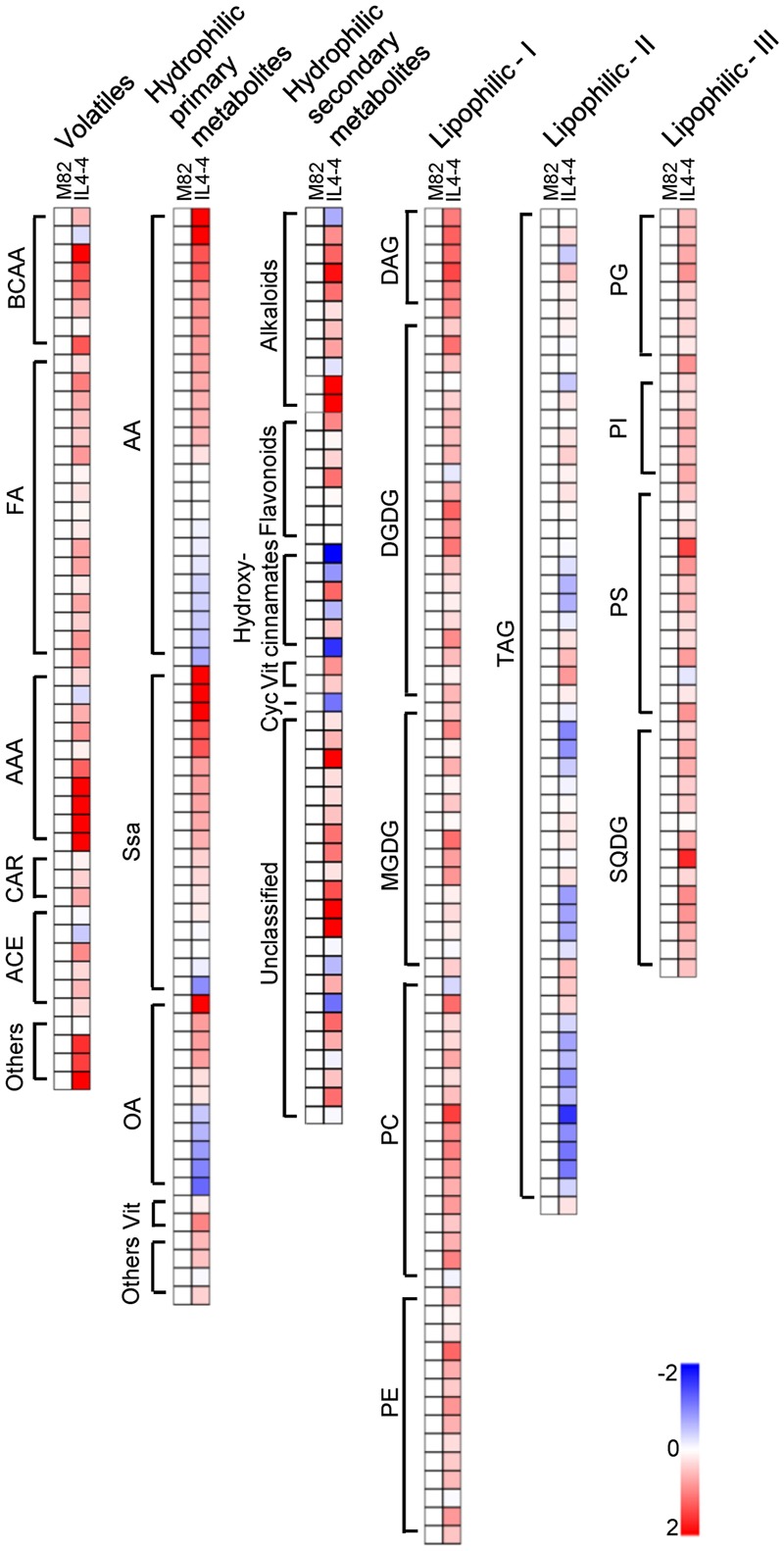
**Heat map of IL4-4 fruit metabolites compared to M82.** BCAA, branched-amino acids; FA, fatty acids; AAA, aromatic-amino acids; CAR, carotenoid; ACE, acetate; AA, amino acids; Ssa, Sugars and sugar alcohols; OA, organic acids; Vit, vitamins; Cyc, cyclitol; Alk, alkaloids; DAG, diacylglycerol; DGDG, digalactosyl-diacylglycerol; MGDG, monogalactosyldiacylglycerol; PC, phosphatidylcholine; PE, phosphatidylethanolamine; TAG, triacylglyceride; PG, phosphatidylglycerol; PI, phosphatidylinositol; PS, phosphatidylserine; SQDG, sulfoquinovosyldiacylglycerol.

To obtain an overview of the IL4-4 fruit metabolome, we quantified a set of metabolites that included VOCs, hydrophilic primary, hydrophilic secondary and lipophilic metabolites by utilizing targeted gas chromatography (targeted GC), gas chromatography-mass spectrometry (GC-MS) and high/ultra-performance liquid chromatography mass spectrometry (HP/UPLC-MS). An overlay heat map shows the metabolite contents in IL4-4 with respect to M82. The fully annotated data set is provided in Supplementary Table S3. Out of 327 quantified fruit metabolites, 185 were altered in the IL4-4 fruit (*p* < 0.05) (**Figure 1**). Chemicals with altered contents included 21 VOCs, 44 hydrophilic primary metabolites, 31 hydrophilic secondary metabolites and 89 lipids (**Table 1**). Consistent with previous results (Tieman et al., 2006), many VOCs were significantly elevated in IL4-4 fruit. These VOCs include chemicals synthesized from multiple independent biosynthetic pathways derived from branched-chain amino acids (BCAAs), fatty acids (FA), and aromatic amino acids (AAA) as well as undefined pathways. These include multiple VOCs associated with consumer liking (Tieman et al., 2012), including 1-nitro-2-phenethane and 3-methyl-1-butanol. Altered hydrophilic primary metabolites included multiple amino acids, organic acids, vitamins and almost all of the measured sugars. Significantly decreased hydrophilic primary metabolites in IL4-4 included multiple amino acids, organic acids and one unknown sugar (**Table 1**). Interestingly, out of 18 quantified sugars and sugar alcohols, 12 were elevated, including the most important contributors to sweetness, glucose, and fructose, while only one unknown sugar was significantly decreased in IL4-4. As key precursors for phenylpropanoids biosynthesis, levels of all three AAA were increased in IL4-4, indicating potential regulators of the shikimate pathway may exist within the *S. pennellii* genomic segment included in IL4-4. In addition, 31 out of 49 hydrophilic secondary metabolites and 89 out of 170 lipids that were quantified were significantly altered (*p* < 0.05) (**Table 1**). These results together indicate a locus or loci at the bottom of *S. pennellii* chromosome 4 regulating multiple independent metabolic pathways during fruit ripening.

**Table 1:** Differential accumulation of fruit metabolites.

Category	Metabolite ID	Ratio
**Volatile organic Compounds**		
Branched-chain	Isovaleronitrile	4.72^∗∗^
Amino acid derived	3-methyl-1-butanol	2.6
	2-methyl-1-butanol	2.16
	2-isobutylthiazole	2.51^∗∗^
Fatty acid derived	1-penten-3-ol	1.22
	1-penten-3-one	2.01^∗∗^
	3-pentanone	1.59^∗∗^
	*cis*-2-penten-1-ol	1.75
	*cis*-3-hexen-1ol	1.64
	heptaldehyde	1.61
	*cis*-4-decenal	1.73^∗∗^
Aromatic-amino acid	1-nitro-2-phenylethane	1.85
derived	Benzyl alcohol	2.36^∗∗^
	Salicylaldehyde	4.74^∗∗^
	Guaiacol	32.29^∗∗^
	Methylsalicylate	168.16^∗∗^
	Eugenol	44.52^∗∗^
Acetate	Hexyl acetate	0.75
Others	2,5-dimethyl-4-methoxy	3.08^∗∗^
	-3(2h)-furanone	
	Isovaleric acid	2.85
	1-nitro-3-methylbutane	5.23^∗∗^
**Hydrophilic primary metabolites**		
Amino acids	Proline	17.78^∗∗^
	Glutmaic acid	6.45^∗∗^
	Pyroglutamic acid	2.54^∗∗^
	Aspartic acid	2.48^∗∗^
	Asparagine	1.85^∗∗^
	Glutamine	1.83^∗∗^
	Cysteine	1.76^∗∗^
	Tryptophan	1.71^∗∗^
	Histidine	1.67^∗∗^
	Tyrosine	1.61^∗∗^
	Ornithine	1.54^∗∗^
	Serine	1.52^∗∗^
	GABA	1.48^∗∗^
	Phenylalanine	1.18
	Glycine	0.75^∗∗^
	Isoleucine	0.74^∗∗^
	Cysteine, s-methyl	0.70^∗∗^
	Valine	0.65^∗∗^
	Alanine	0.58^∗∗^
Sugars and sugar alcohols	Glucoheptonic acids, 4-lacton	5.77^∗∗^
	Trehalose,α,α	5.18^∗∗^
	Galactinol	4.93^∗∗^
	Isomaltose	2.62^∗∗^
	Raffinose	2.48^∗∗^
	Sugar204	1.68^∗∗^
	Glucose	1.68^∗∗^
	Threitol	1.65^∗∗^
	Sucrose	1.59^∗∗^
	Fructose	1.53^∗∗^
	Rhamnose	1.28^∗∗^
	Glucose, 1,6-anhydro, β	1.15
	Sugar1_9.34	0.46^∗∗^
Organic acids	Glucaric acid	1.71^∗∗^
	Citric acid	1.7
	Glucornic acid	1.68^∗∗^
	Glyceric acid	0.69
	Pyruvic acid	0.61^∗∗^
	Succinic acid	0.51^∗∗^
	Malic acid	0.43^∗∗^
	Fumaric acid	0.36^∗∗^
Vitamins	Inositol, myo	1.95^∗∗^
Others	Indole_rt.8.78	1.47^∗∗^
	Urea	1.39
	Glycerol	1.29^∗∗^
**Hydrophilic secondary metabolites**		
Flavonoids	Putative_flavonoid_I	1.94^∗∗^
	Putative_flavonoid_II	2.17^∗∗^
Hydroxycinnamates	Caffeoyl-hexose-II	2.31^∗∗^
	Caffeoyl-hexose-II	0.56^∗∗^
	Caffeoylquinic acid-II	1.39^∗∗^
	Dicaffeoylquinic acid	0.33
Alkaloids	Putative_glycoalkaloid	1.87^∗∗^
	Putative_glycoalkaloid	3.62^∗∗^
	Putative_glycoalkaloid	2.33^∗∗^
	Esculeoside related	2.19^∗∗^
	Esculeoside related	1.42^∗∗^
	Esculeoside related	1.68^∗∗^
	Calystegine A3	4.21^∗∗^
	Calystegine B2	4.08^∗∗^
Vitamins	Nicotinamide	1.81^∗∗^
	Nicotinic acid	1.34
Cyclitol	Quinic acid, 3-caffeoy, *trans*	0.39
Unclassified	Propanoic acid-hexose	1.51
Unknown	Unclassified	1.39
	Unclassified	2.16^∗∗^
	Unclassified	2.11
	Unclassified	2.6
	Unclassified	6.2
	Unclassified	5.32^∗∗^
	Unclassified	0.68
	Unclassified	1.57
	Unclassified	0.46^∗∗^
	Unclassified	2.28^∗∗^
	Unclassified	1.57^∗∗^
	Unclassified	2.21^∗∗^
	Unclassified	4.52^∗∗^
**Lipids**		
Diacylglycerol (DAG)	DAG 34:2	2.04^∗∗^
	DAG 34:3	2.37^∗∗^
	DAG 36:2	2.24^∗∗^
	DAG 36:3	2.73^∗∗^
	DAG 36:4	2.04^∗∗^
	DAG 36:5	1.90^∗∗^
Digalactosyl-Diacylglycerol	DGDG 32:0	1.34
(DGDG)	DGDG 32:1 (1)	2.17^∗∗^
	DGDG 32:3 (2)	1.45^∗∗^
	DGDG 34:3	1.52^∗∗^
	DGDG 34:4 (1)	2.34^∗∗^
	DGDG 34:4 (2)	1.75^∗∗^
	DGDG 34:5	2.12^∗∗^
	DGDG 36:3 (2)	1.89^∗∗^
	DGDG 36:6	1.48^∗∗^
Monogalactosyl-	MGDG 32:1	1.91
diacylglycerol (MGDG)	MGDG 34:1	1.54
	MGDG 34:4 (1)	2.22^∗∗^
	MGDG 36:1	1.77^∗∗^
	MGDG 36:6	1.31^∗∗^
Phosphatidylcholine (PC)	PC 32:1	2.23^∗∗^
	PC 34:1	1.60^∗∗^
	PC 34:2	1.2
	PC 34:3	1.42^∗∗^
	PC 34:4 (1)	2.87^∗∗^
	PC 34:4 (2)	1.85^∗∗^
	PC 34:5	2
	PC 36:1	1.73^∗∗^
	PC 36:2	1.55^∗∗^
	PC 36:3	1.73
	PC 36:4	1.35^∗∗^
	PC 36:5	1.54^∗∗^
	PC 36:6	1.99^∗∗^
Phosphatidyl	PE 34:1	1.48
Ethanolamine (PE)	PE 34:4	2.31^∗∗^
	PE 36:1	1.57^∗∗^
	PE 36:2	1.34^∗∗^
	PE 36:3 (1)	1.78
	PE 36:3 (2)	1.54^∗∗^
	PE 36:4	1.21
	PE 36:5	1.34^∗∗^
	PE 36:6	1.46
	PE 38:3	1.76^∗∗^
Triacylglyceride (TAG)	TAG 48:2 (2)	1.39
	TAG 52:6	1.46^∗∗^
	TAG 52:7	1.76
	TAG 56:0	1.17
	TAG 56:2	0.52
	TAG 56:4	0.56
	TAG 56:6	1.43
	TAG 56:7	1.37^∗∗^
	TAG 58:2	0.54
	TAG 58:4	0.48^∗∗^
	TAG 58:5	0.64^∗∗^
	TAG 58:6	0.12^∗∗^
Phosphatidylglycerol (PG)	PG 32:0	1.43
	PG 32:1	1.47^∗∗^
	PG 34:0	1.59
	PG 34:1 (1)	1.80^∗∗^
	PG 34:2 (1)	1.30^∗∗^
	PG 34:4	1.80^∗∗^
Phosphatidylinositol (PI)	PI 34:1	1.26
	PI 34:2	1.20^∗∗^
	PI 34:3	1.48^∗∗^
	PI 36:4	1.50^∗∗^
	PI 36:5	1.40^∗∗^
	PI 36:6	1.58
Phosphatidylserine (PS)	PS 38:0	1.34^∗∗^
	PS 38:1	1.08
	PS 38:2	1.33^∗∗^
	PS 38:3	2.75^∗∗^
	PS 40:0	1.80^∗∗^
	PS 40:1	1.38^∗∗^
	PS 40:2	1.49^∗∗^
	PS 40:3	1.22^∗∗^
	PS 40:4	1.23^∗∗^
	PS 40:5	1.72^∗∗^
	PS 42:1 (1)	0.85
Sulfoquinovosyl-	SQDG 32:0	1.28^∗∗^
Diacylglycerol (SQDG)	SQDG 32:2 (1)	1.57^∗∗^
	SQDG 32:4	1.32
	SQDG 34:1	1.35
	SQDG 34:3	1.60^∗∗^
	SQDG 34:4	3.20^∗∗^
	SQDG 36:3 (1)	1.87
	SQDG 36:3 (2)	1.80^∗∗^
	SQDG 36:4	1.54^∗∗^
	SQDG 36:5	1.40^∗∗^
	SQDG 36:6	1.4

*Ratio indicates fold change of metabolites from ripe fruits of IL4-4 compared to M82. Only metabolites with at least a significant difference of *p* < 0.05 are listed. Double asterisks indicate significant differences of *p* < 0.01.*

### Transcriptomic Analysis of IL4-4

**FIGURE 2:**
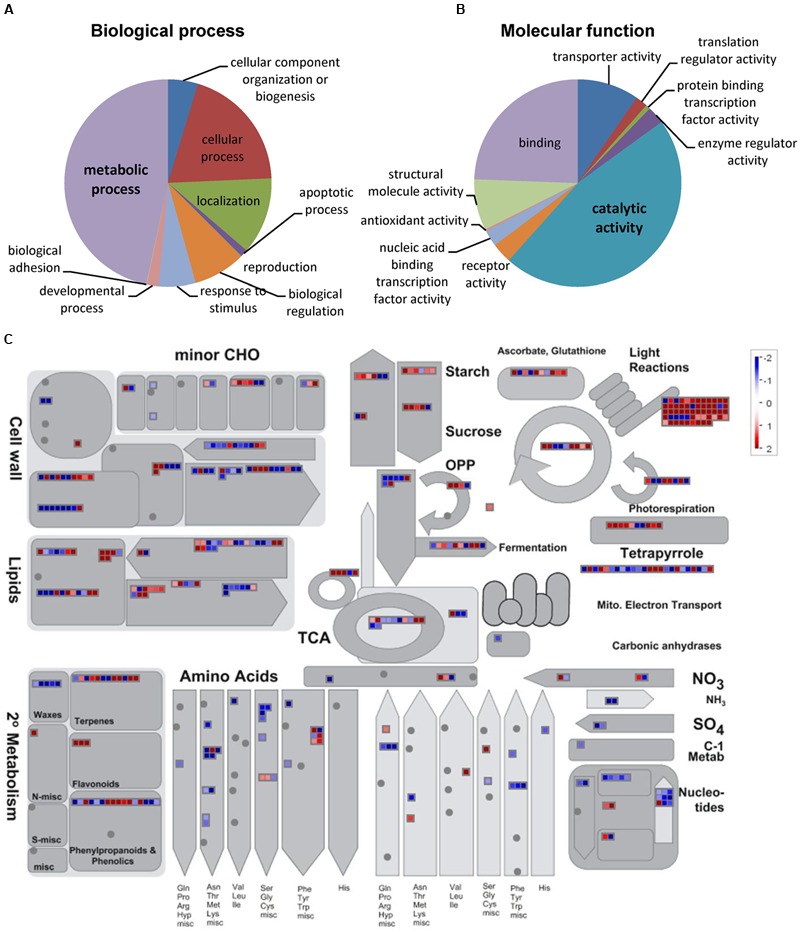
**Functional classification of DEGs between IL4-4 and M82.** Distribution of GO terms for biological process **(A)** and molecular function **(B)** conducted with the PANTHER web tool. **(C)** Overview of the effect of IL4-4 regulated expression changes on metabolism of fruits with MapMan software (Metabolism_overview panel). MapMan functional categories and Log2 fold change values are represented. Significantly increased and decreased transcripts are shown in red and blue, respectively. A dot represents the log2 of the RPKM ratio of a transcript between IL4-4 and control M82.

To gain insight into the molecular mechanisms underlying the observed metabolite changes, gene expression in ripe pericarp tissues of IL4-4 and M82 was determined using RNA-Seq. A total of 26,147 expressed genes were detected in IL4-4 and M82 (Supplementary Table S5). To identify DEGs, we determined the significance of difference in transcript contents. Using cutoff criteria of ≥2-fold difference in transcript and adjusted *P*-value < 0.05, we identified 2307 more abundant transcripts and 1635 less abundant transcripts in IL4-4 fruit tissue compared to M82 (Supplementary Table S6). Next, we performed GO enrichment analysis with the DEGs to identify the major gene groups whose transcript abundance was altered in IL4-4. Due to the major alterations in biochemical composition in IL4-4, we examined the GO term distribution within biological process and molecular function categories (**Figures 2A,B**). GO analysis showed that the largest groups in biological process and molecular function were metabolic process (47.4%) and catalytic activity (45%), respectively.

In order to further investigate the potential roles of DEGs in metabolism, we generated a snapshot of these genes over the main metabolic pathways facilitated by MapMan. An overview of the map suggested a global transcriptional regulation of metabolism-associated genes by IL4-4 (**Figure 2C**). The mapped DEGs are involved in main primary and secondary metabolic biosynthetic processes such as sugar, acid, amino acid, phenylpropanoid, and lipid metabolism. Notably, light reaction-associated genes were significantly more abundant. This pattern suggests a possible increase in photosynthesis in IL4-4 fruit, which in turn could provide precursors for multiple metabolic pathways.

**FIGURE 3:**
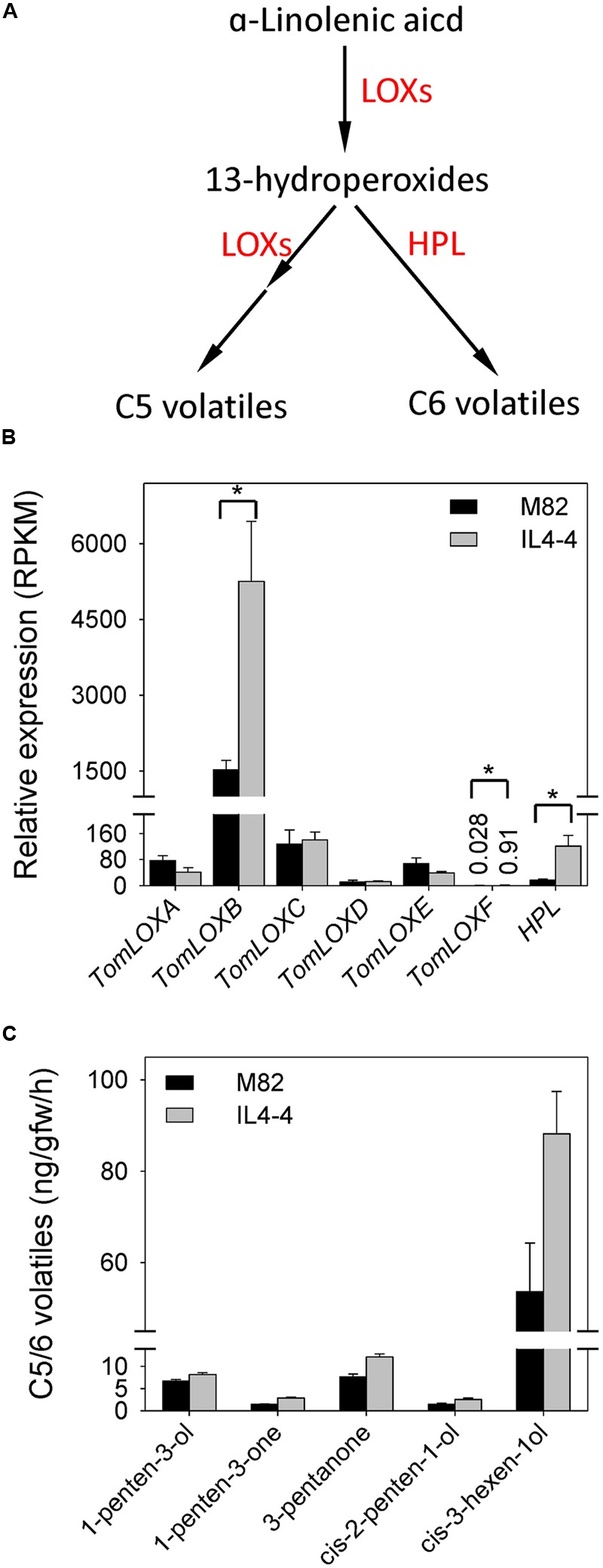
**Levels of volatiles and transcripts in the fatty acid pathway in IL4-4 and M82. (A)** Schematic representation of the C5 and C6 volatile biosynthetic pathway based on Shen et al. (2014). HPL, hydroperoxide lyase; LOXs, lipoxygenases. **(B)** Expression of *LOX* f and *HPL* genes in ripe tissue of IL4-4 and M82. Ratio (±SE) was calculated by the average RPKM value. An asterisk indicates RPKM ration change >2 and adjusted *p* < 0.05. **(C)** levels of C5 and C6 volatiles (±SE) significantly different (*p* < 0.05, *n* > 5) between IL4-4 and M82.

To better understand the relationship between transcription and metabolite synthesis in IL4-4, we examined transcript content of genes encoding key enzymes related to FA metabolism and the shikimate-phenylpropanoid pathway. 13-lipoxygenases (13-LOXs) and hydroperoxide lyase (HPL) are the main enzymes catalyzing conversion of C18 polyunsaturated FAs to C5 and C6 volatiles in tomato fruit (**Figure 3A**). We observed significantly higher levels of *TomLOXB* (Solyc01g099190), *TomLOXF* (Solyc01g006560) and *HPL* (Solyc07g049690) transcripts in IL4-4 fruit tissue while other members of the *13-LOX* family remained unchanged (**Figure 3B**). Increased transcript abundance of these genes is consistent with the higher levels of C5 and C6 volatiles in IL4-4 fruit (**Figure 3C**).

**FIGURE 4:**
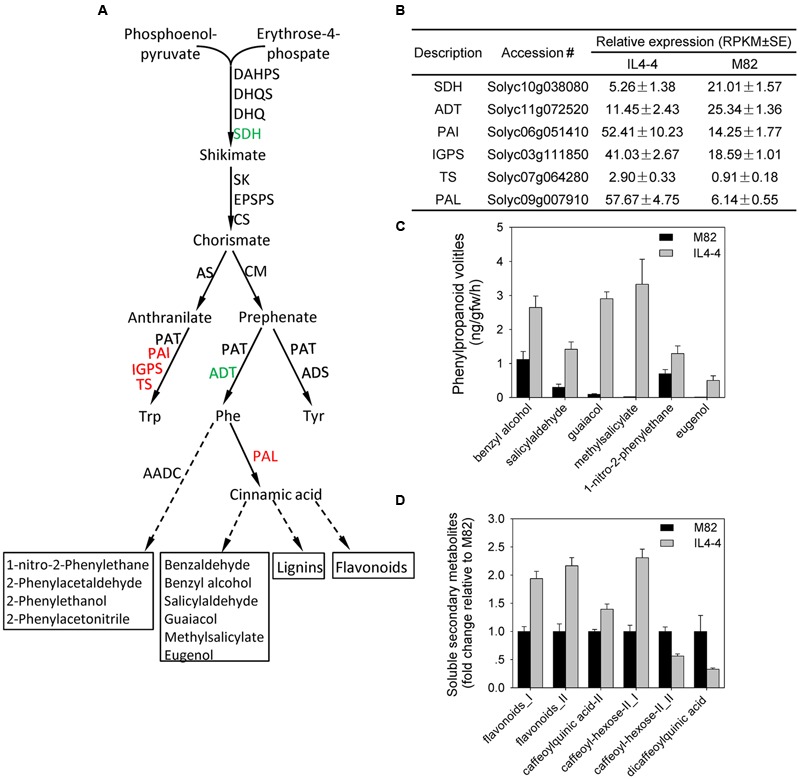
**Changes of metabolites and transcripts in the shikimate-phenylpropanoid pathway in IL4-4 compared to M82. (A)** Shikimate and phenylpropanoid pathways. Genes whose transcripts were significantly elevated or reduced in IL4-4 are indicated in red and green, respectively. Dashed arrows represent multiple enzymatic steps. ADT, arogenate dehydratase; ADS, arogenate dehydrogenase; AADC, aromatic amino acid decarboxylase; CM, chorismate mutase; CS, chorismate synthase; C4H, cinnamate-4-hydroxylase; CHS, chalcone synthase; 4CL, 4-coumarate CoA ligase; DAHPS, 3-deoxy-D-arabino-2-heptulosonate 7-phosphate synthase; DHQS, 3-dehydroquinate synthase; EPSPS, 5-enolpyruvylshikimate-3-phosphate synthase; EGS, eugenol synthase; IGPS, indole-3-glycerol phosphate synthase; PAI, anthranilate isomerase; PAL, phenylalanine ammonia-lyase; PAR, phenylacetaldehyde reductase; SK, shikimate kinase; SQH, shikimate dehydrogenase; TS, tryptophan synthase. **(B)** RPKM values of DEGs in the shikimate and phenylpropanoid pathways. **(C)** Levels (±SE) of significantly altered phenylpropanoid volatiles (*p* < 0.05). **(D)** Relative levels of hydrophilic phenylpropanoids. Results are presented as fold change relative to M82 (*n* > 5).

We observed significant increases in AAAs as well as nutrition and flavor contributing phenylpropanoids and glycoalkaloids in IL4-4. Therefore, we examined the transcript levels of genes encoding key steps in the shikimate and phenylpropanoid pathways (**Figure 4A**). The transcripts associated with most genes in the shikimate pathway were not significantly different while there was a significant decrease in shikimate dehydrogenase (Solyc10g038080) in IL4-4. Transcripts encoding three genes in the tryptophan synthesis pathway (*PAI*: anthranilate isomerase, Solyc06g051410; *IGPS*: indole-3-glycerol phosphate synthase, Solyc03g111850; *TS*: tryptophan synthase, Solyc07g064280) were elevated in IL4-4, while *ADT* (arogenate dehydratase: Solyc11g072520) in the phenylalanine pathway was reduced. In addition, we observed significantly higher transcript contents of *PHENYLALANINE AMMONIA-LYASE* (*PAL*: Solyc09g007910), which catalyzes the first step in phenylpropanoid synthesis from L-phenylalanine (**Figure 4B**). A previous study suggested that expression of this *PAL* is the primary determinant of commitment to phenylpropanoid synthesis (Xie et al., 2016). Therefore, higher Solyc09g007910 transcript in IL4-4 could explain the higher levels of multiple phenylpropanoids as well as several Phe-derived volatiles in IL4-4 compared to M82 (guaiacol, methylsalicylate and eugenol) (**Figures 4C,D**). Taken together, these results suggest that genetic factors within IL4-4 regulate metabolite levels via their influence on transcription of genes involved in multiple biosynthetic pathways.

### Fine Mapping of the QTLs Controlling Metabolic Changes of IL4-4 Fruit

**FIGURE 5:**
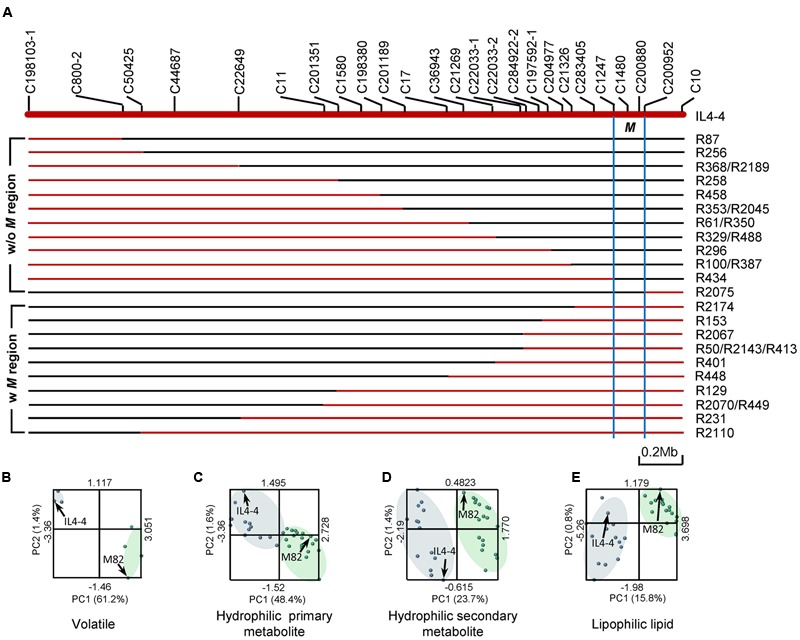
**Association of metabolic phenotypes with sub-ILs. (A)** Schematic representation of sub-ILs derived from IL4-4. Red and blue lines represent segments of *S. pennellii* and *S. lycopersicum*, respectively. Genomic region *M* was defined as the area between markers C1247 and C200952, indicated by blue lines. The starts and ends of the sub-ILs were defined by the molecular markers listed above (Supplementary Table S6). **(B–E)** PLS-EDA analysis of metabolite contents of sub-ILs and their parent lines. Phenotypic similarity is indicated by the distance between each point. Blue dots represent IL4-4 and lines with the M region; green dots represent M82 and lines without the M region. IL4-4 and M82 are indicated with arrows. **(B)** Volatile metabolites; **(C)** Hydrophilic primary metabolites; **(D)** Hydrophilic secondary metabolites; **(E)** Lipophilic metabolites.

IL4-4 includes a genomic region of ∼3.4Mb (Chitwood et al., 2013). To fine map the loci affecting metabolic traits in IL4-4 fruit, we generated a set of sub-ILs by screening an F2 population of IL4-4 crossed to M82. Mapping of the recombinant lines was accomplished using molecular markers developed from alignment of the *S. lycopersicum* and *S. pennellii* genomes (**Figure 5A**). VOCs were collected from fruits of five selected sub-ILs (R2174, R401, R100, R2075, and R434) and the parents, M82 and IL4-4, while additional metabolite data were collected from all sub-ILs shown in **Figure 5A**. Partial least squares enhanced discriminant analysis (PLS-EDA) models were generated based on the metabolome data collected from the sub-ILs (**Figures 5B–E**). The distribution patterns of different lines with and without the *M* genomic region suggest that the overall metabolic traits of all lines can be divided to two main clades, IL4-4-like and M82-like. Sub-ILs that contain *S. pennellii* genomic region *M* (defined by marker C2147 and C200952) are closer to IL4-4, while lines without the *M* region resemble M82. Thus, the *S. pennellii M* genome fragment contains the major locus affecting a wide range of metabolic changes in tomato fruit.

**FIGURE 6:**
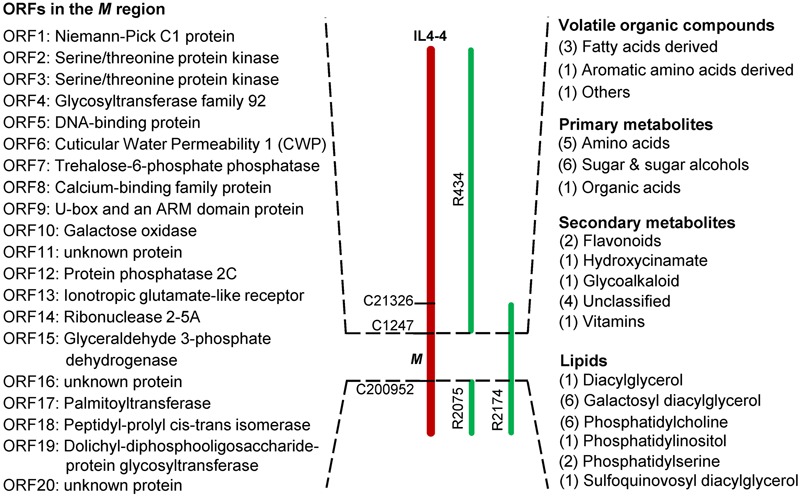
**Metabolites significantly altered in lines containing the *M* region of IL4-4.** Numbers of altered metabolites within a class are indicated in brackets. Putative open reading frames (ORFs) within the *M* genome region are listed in order.

**FIGURE 7:**
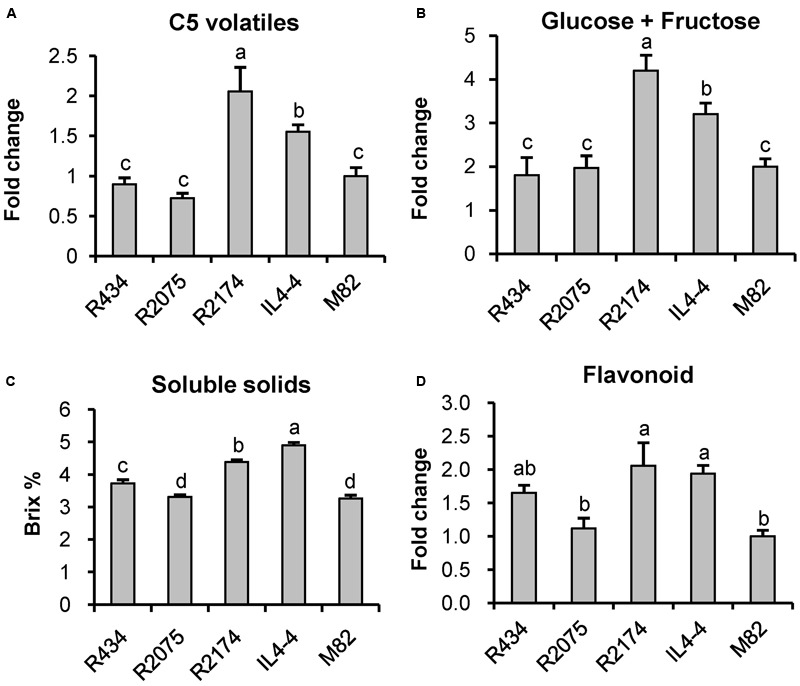
**Fruit quality associated features regulated by the *M* genomic region. (A)** C5 volatiles include 1-penten-3-ol, 1-penten-3-one, 3-pentanone, *E*-2-pentenal, 1-pentanol and *Z*-2-penten-1-ol. **(B)** glucose plus fructose. **(C)** soluble solids. **(D)** flavonoids. Letters represent significant difference among the five genotypes using ANOVA followed by a Newman–Keuls test (*p* < 0.01).

As shown in **Table 1**, we identified 185 significantly altered metabolites (*p* < 0.05). To further understand how the *M* genomic region regulates fruit quality, we reexamined all of these metabolites in all of the lines for significant effects using ANOVA. Using a statistical threshold of *p* < 0.01, we identified 72 metabolite QTLs in IL4-4. Of these QTLs, 43 were located within the *M* region (Supplementary Table S4; **Figure 6**). QTLs within this region affect metabolites synthesized from a wide range of both primary and secondary metabolism pathways. Interestingly, QTLs correlated with fructose, glucose, soluble solids (brix) and C5 volatile contents are located within this small segment, all traits likely to affect consumer taste preferences (**Figures 7A–C**). There are also major effects on potential nutrition-associated metabolites, including flavonoids (**Figure 7D**). To address the effects of these chemical alterations on flavor quality, we conducted a consumer panel where individuals rated fruits from M82, IL4-4, and sub-ILs. Consumers significantly preferred IL4-4 and a sub-IL containing the *M* region (R2174) over M82 or sub-ILs absent the *M* region (Supplementary Figure S1).

There are 20 annotated genes within the *M* region of the *S. lycopersicum* and *S. pennellii* genomes (**Table 2**; **Figure 6**). In the red fruit pericarp tissue, transcript levels of only three of these genes were significantly altered between IL4-4 and M82: *ORF2* (Serine/threonine protein kinase), *ORF10* (Galactose oxidase/kelch repeat superfamily protein) and *ORF17* (Palmitoyltransferase). There were no premature stop codons in any open reading frames of the genes in either ortholog. To investigate the possible effects of amino acid sequence substitutions on the metabolic phenotype in IL4-4, a comparison of protein sequences of the 20 candidate genes from *S. pennellii* and *S. lycopersicum* located in the mapped *M* genome region was performed (Supplementary Table S7). As predicted by the SIFT analysis, amino acid sequence polymorphisms of 13 candidates are not likely to be functionally significant, alteration of amino acids in five candidates likely confer a functional change, while the remainder did not show a clear effect. Notably, *CWP1* was previously reported to cause a dehydration phenotype in ripe fruit (Hovav et al., 2007). That skin phenotype is manifested in sub-ILs containing the *M* region (Supplementary Figure S2). However, the water loss phenotype associated with CWP1 cannot explain the entire effect on metabolites in IL4-4 fruit. For example, while many metabolites are higher, as might be expected in a fruit with less water content, levels of multiple organic acids are reduced in IL4-4. Therefore, further screening of recombinant events in this *M* region combined with transgenic alterations will be necessary to map the gene(s) responsible for altered metabolite biosynthesis.

**Table 2:** List of candidate genes in the *M* region.

Number	Molecular marker	Putative ORF	Predicted protein function	Expression (RPKM)	
				M82	IL4-4
(1)	C1247	ORF 1	Niemann-Pick C1 protein	2.22	1.56
(2)		ORF 2^∗^	Serine/threonine protein kinase	1.47	0.1
(3)		ORF 3	Serine/threonine protein kinase	38.25	42.69
(4)		ORF 4	Glycosyltransferase family 92	0.06	0.01
(5)		ORF 5	DNA-binding protein	1.36	1.33
(6)	C91665	ORF 6	Cuticular Water Permeability 1 (CWP)	0	0.16
(7)	C1480	ORF 7	Trehalose-6-phosphate phosphatase	0	0.01
(8)		ORF 8	Calcium-binding family protein	20.76	15.98
(9)		ORF 9	U-box and an ARM domain protein	10.73	18.29
(10)		ORF 10^∗^	Galactose oxidase	2.14	7.73
(11)		ORF 11	Unknown protein	0.37	0.98
(12)		ORF 12	Protein phosphatase 2C	26.18	24.5
(13)		ORF 13	Ionotropic glutamate-like receptor	1.29	0.83
(14)		ORF 14	Ribonuclease 2-5A	8.48	6.74
(15)	c200880	ORF 15	Glyceraldehyde 3-phosphate dehydrogenase	0.24	0.35
(16)		ORF 16	Unknown protein	56.7	72.88
(17)		ORF 17^∗^	Palmitoyltransferase	3.16	0.59
(18)		ORF 18	Peptidyl-prolyl *cis*–*trans* isomerase	0.25	0.22
(19)		ORF 19	Dolichyl-diphosphooligosaccharide-protein glycosyltransferase	48.33	28.99
(20)	c200952	ORF 20	Unknown protein	39.8	56.31

*Asterisks indicate differential expression between IL4-4 and M82 (cut-off of fold change >2, adjusted *p* < 0.05).*

## Discussion

### The Genetic Basis of Fruit Biochemical Diversity in IL4-4

This study provides a comprehensive analysis of the metabolite composition of fruit from M82 and the highly chemically variant line, IL4-4, as well as sub-ILs. Multiple studies have identified metabolite QTLs in introgression populations, including those derived from *S. pennellii* (Schauer et al., 2006; Tieman et al., 2006; Mathieu et al., 2009; Alseekh et al., 2015). Here, we describe an extensive metabolite analysis of IL4-4, identifying 44 hydrophilic primary metabolites, 89 lipids, 31 hydrophilic secondary metabolites and 21 VOCs that are significantly altered relative to M82. The effect on such a large set of metabolites indicates the potential of this small locus to have a major impact on tomato fruit quality.

High-resolution fine mapping studies with tomato populations to identify loci regulating multiple fruit quantitative traits have been performed (e.g., Frary et al., 2000; Fridman et al., 2004). Yates et al. (2004) mapped a series of phenotypes including brix, fruit weight, stem scar, lycopene content, reticulation, and fruit shape to a three centimorgan segment at the bottom of IL4-4 by analysis of a set of sub-ILs of IL4-4. Hovav et al. (2007) demonstrated that the “reticulation” phenotype is a result of mis-expression of a *CWP1* gene with map-based cloning from *S. habrochaites* sub-ILs. Neither group provided information on an extensive set of metabolites. By fine mapping a set of sub-ILs derived from IL4-4, we identified a segment of 200 kb that significantly alters the contents of more than 40 chemicals (*p* < 0.01). Localization of the reticulated peel phenotype caused by CWP1 (Hovav et al., 2007) to the *M* region might explain some of the altered metabolites. Hovav et al. (2007) did not examine metabolites in fruit flesh and the transgenic lines reported are not available. Although *CWP1* expression is limited to the cuticle of immature fruits (Hovav et al., 2007), higher metabolite content in mature fruit flesh could be the consequence of dehydration caused by the micro-fissured fruit cuticle. For example, contents of proline were significantly increased, possibly correlating with water loss. Water loss could also affect soluble solids content. However, water loss cannot be the sole explanation for the *M* region phenotype as levels of a large portion of organic and amino acids were decreased. Separately, we note the existence of a *TREHALOSE-6-PHOSPHATE PHOSPHATASE* (*T6PP*) gene in the *M* region. Trehalose, which is regulated by T6PP, has been proposed to be a central signaling sugar that impacts sucrose content (Nuccio et al., 2015). Altered sucrose metabolism, in turn, could have major effects on many fruit metabolites. There were three genes whose transcripts were significantly altered in IL4-4 (**Table 2**). It is possible that altered expression of one or more of these genes could cause at least some of the observed metabolic changes.

In general, GO enrichment analysis of the DEGs suggested a major impact on genes involved in metabolic processes and catalytic activity in ripe IL4-4 fruit tissue. The DEGs were distributed among multiple distinct metabolic pathways. We were able to identify genes with important functions in FA and phenylpropanoid pathways whose transcripts were more abundant in IL4-4. In the case of FAs, multiple *LOX* transcripts as well as *HPL* were more abundant. For phenylpropanoids, the transcript encoding PAL (Solyc09g007910) was significantly higher in IL4-4. This transcript was previously shown to be significantly up-regulated in transgenic tomato fruits over-expressing the MYB transcription factor PhODO1 (Dal Cin et al., 2011). Those plants also contain substantially higher levels of many phenylpropanoid compounds (Xie et al., 2016). Notably, the *S. pennellii M* region defined here does not contain any transcription factors. Therefore, the effects on gene products involved in these metabolic pathways must be indirect. Higher levels of transcripts in light reaction-associated genes could impact photosynthetic capacity, having an effect on many metabolites.

In summary, we have mapped a major metabolite QTL to a genome region encoding only 20 genes. This locus has a broad effect on multiple metabolic pathways, many contributing to overall fruit flavor and nutritional quality. Further work is necessary to identify the causative gene(s) and the mechanism of action. Although the skin reticulation phenotype is not itself desirable, it may be possible to eliminate or minimize this effect while maximizing the overall effects on fruit metabolite contents.

## Author Contributions

ZL, HK, AF, JG, and ZF organized and coordinated aspects of the research in their respective laboratories. ZL, SA, YB, DT and YZ performed experiments and data analysis. ZL, SA, HK, and AF wrote the manuscript.

## Conflict of Interest Statement

The authors declare that the research was conducted in the absence of any commercial or financial relationships that could be construed as a potential conflict of interest.

## Footnotes

1 www.agilent.com

2 http://www.thermofisher.com

3 http://www.genedata.com

4 http://www.ebi.ac.uk/Tools/msa/clustalw2/

5 http://sift.jcvi.org/www/SIFT_BLink_submit.html

6 http://pantherdb.org/
