# Supplementary material for: Identification of a Solanum pennellii Chromosome 4 Fruit Flavor and Nutritional Quality-Associated Metabolite QTL
Source: Front Plant Sci. 2016 Nov 9;7:1671. doi: 10.3389/fpls.2016.01671 (PMC5101573; doi:10.3389/fpls.2016.01671)
Supplement: Supplementary file 8 [file Data_Sheet_1.DOCX]

Supplementary Material

Characterization of *Solanum pennellii* Chromosome 4 Fruit Quality-Associated Metabolite QTLs

**Zhongyuan Liu^1#^, Saleh Alseekh^2#^, Yariv Brotman^2,3^, Yi Zheng^4^, Zhangjun Fei^4^, Denise M. Tieman^1^, James J. Giovannoni^4^, Alisdair R. Fernie^2^, Harry J. Klee^1,*^**

*** Correspondence:** Harry J. Klee: [hjklee@ufl.edu](mailto:hjklee@ufl.edu)

# Supplementary Figures and Tables

## Supplementary Figures

**
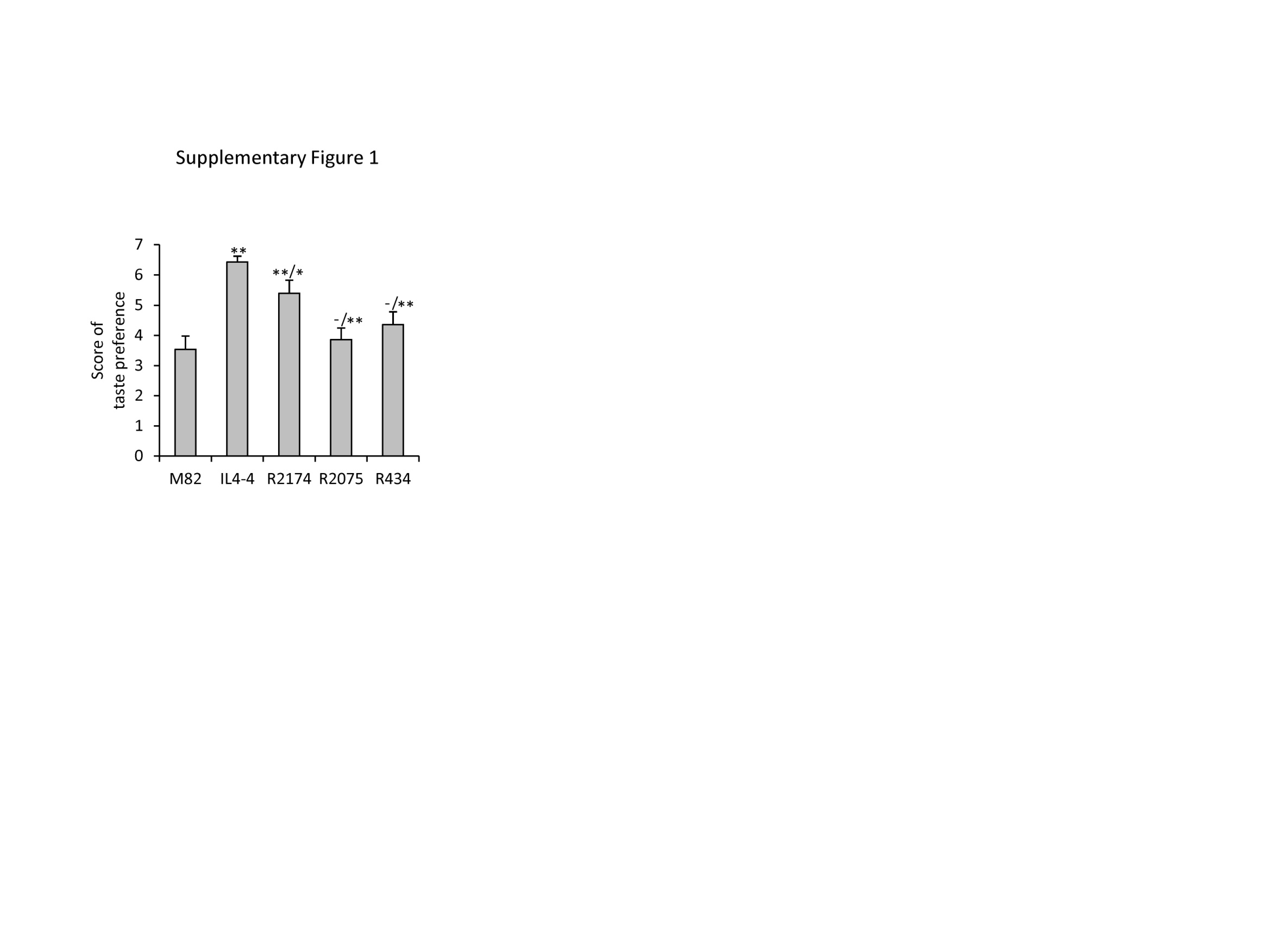
**

**Supplementary Figure 1.** Taste preference scores (±SE) of fruits from M82, IL4-4 and sub-ILs with (R2174) or without the *M* region (R2075, R434). The significant difference of scores of each sub-IL relative to M82 and IL4-4 is indicated (M82/IL4-4). * p < 0.05; ** p < 0.01.


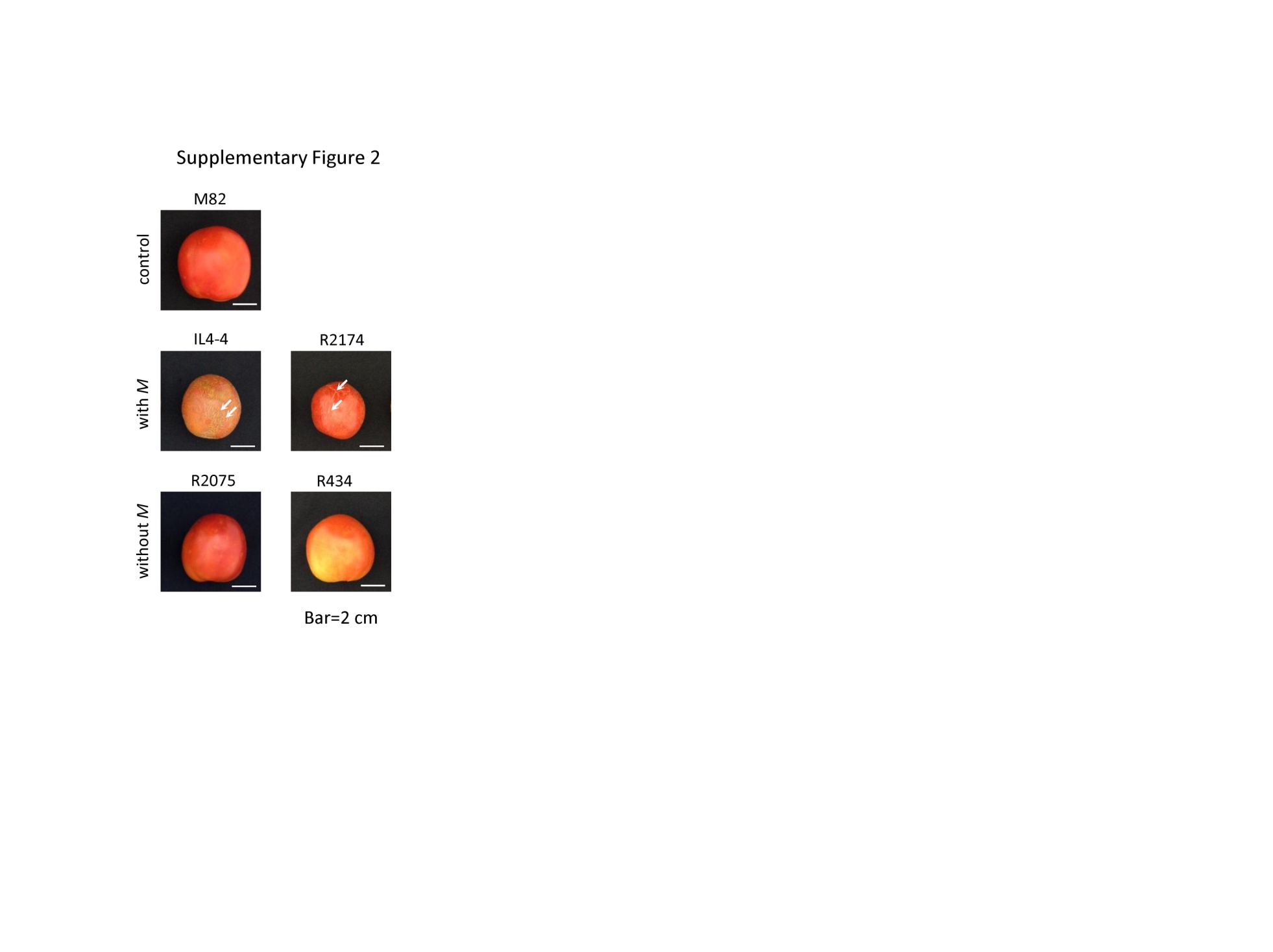


**Supplementary Figure 2.** Fruit reticulation phenotype in lines with and without the *M* genome region. Bar = 2 cm. White arrows point to the micro-fissuring of the fruit cuticle.

## Supplementary Tables

**Supplementary Table 1.** Physical maps of sub-NILs and information of molacular markers used in this study.

**Supplementary Table 2.** Validation of sugar and acid metabolites contents in IL4-4 and M82 fruits harvested from different growth conditions. Data was expressed as fold change of IL4-4/M82. Asterisk indicate significant differences of p<0.05, with Student *t-*test (p<0.01 indicated by double asterisks).

**Supplementary Table 3.** Metabolite profiles of each line.

**Supplementary Table 4.** QTL mapping result file.

**Supplementary Table 5.** RNA-seq data of IL4-4 and M82.

**Supplementary Table 6.** Differentially expressed genes in IL4-4.

**Supplementary Table 7.** Protein sequence comparison of the candidates in the mapped *M* genome region.
